# Reactive oxygen species are required for zoledronic acid-induced apoptosis in osteoclast precursors and mature osteoclast-like cells

**DOI:** 10.1038/srep44245

**Published:** 2017-03-10

**Authors:** Ta-Wei Tai, Ching-Yu Chen, Fong-Chin Su, Yuan-Kun Tu, Tsung-Ting Tsai, Chiou-Feng Lin, I.-Ming Jou

**Affiliations:** 1 Department of Orthopedics, National Cheng Kung University Hospital, College of Medicine, National Cheng Kung University, Tainan 701, Taiwan; 2 Department of Biomedical Engineering, College of Engineering, National Cheng Kung University, Tainan 701, Taiwan; 3 Department of Orthopedics, E-Da Hospital, Kaohsiung 824, Taiwan; 4 Department of Microbiology and Immunology, School of Medicine, College of Medicine, Taipei Medical University, Taipei 110, Taiwan; 5 Graduate Institute of Medical Sciences, College of Medicine, Taipei Medical University, Taipei 110, Taiwan

## Abstract

Inhibiting osteoclasts and osteoclast precursors to reduce bone resorption is an important strategy to treat osteoclast-related diseases, such as osteoporosis, inflammatory bone loss, and malignant bone metastasis. However, the mechanism by which apoptosis is induced in the osteoclasts and their precursors are not completely understood. Here, we used nitrogen-containing bisphosphonate zoledronic acid (ZA) to induce cell apoptosis in human and murine osteoclast precursors and mature osteoclast-like cells. Caspase-3-mediated cell apoptosis occurred following the ZA (100 μM) treatment. Reactive oxygen species (ROS) were also generated in a time-dependent manner. Following knock-down of the p47^phox^ expression, which is required for ROS activation, or co-treatment with the ROS inhibitor, N-acetyl-L-cysteine, ZA-induced apoptosis was significantly suppressed in both osteoclast precursors and mature osteoclast-like cells. The ROS-activated mitogen-activated protein kinases pathways did not trigger cell apoptosis. However, a ROS-regulated Mcl-1 decrease simultaneously with glycogen synthase kinase (GSK)-3β promoted cell apoptosis. These findings show that ZA induces apoptosis in osteoclast precursors and mature osteoclast-like cells by triggering ROS- and GSK-3β-mediated Mcl-1 down-regulation.

Osteoporosis is caused by an imbalance of osteoblasts and osteoclasts. Specifically, the bone formation of functioning osteoblasts is suppressed, and osteoclasts are over activated for bone resorption^1^. Clinically, osteoporosis is characterized by low bone mineral density and an abnormal bony structure and quality. Osteoporosis leads to decreased bone strength and increased susceptibility to fractures^2^. Osteoporosis can cause a great deal of disability and may increase the risk of death, especially when hip fractures occur^3^.

Bisphosphonates, which are pyrophosphate analogues utilized as bone-specific anti-resorptive agents, are the most common agents for the treatment of osteoporosis. These agents act by inhibiting osteoclasts^4^. However, poor compliance with the oral form of bisphosphonates is often observed because of upper gastrointestinal tract irritation and the strict dosing schedule required^5^. Therefore, new, once-a-year intravenous drugs, such as zoledronic acid (ZA), have been developed to allow dosing at much longer intervals to improve therapy compliance^6^. Treatment with ZA results in higher trabecular volume, higher trabecular numbers, and decreased separation^7^. A large international clinical trial demonstrated that patients treated with ZA show significant improvements in low bone mineral density and bone metabolism markers. Treatment with ZA reduces the risk of vertebral fracture by 70% and hip fracture by 41% over 3 years relative to placebo^8^.

Pharmacologically, ZA inhibits the farnesyl diphosphate-mediated mevalonate pathway, thereby inhibiting osteoclast proliferation and inducing apoptotic cell death in osteoclasts^4^,^9^. However, the intracellular pro-apoptotic pathway is still unknown. Previous studies have shown that the use of ZA may significantly enhance apoptosis by elevating reactive oxygen species (ROS) levels in prostate carcinoma, multiple myeloma, and salivary adenoid cystic carcinoma cell models^10^,^11^. ROS are reactive molecules containing oxygen, such as superoxide anion (O^2−^) and hydrogen peroxide^12^ and also nitric oxide. ROS are normal by-products of cellular metabolism, but are hazardous in some situation such as aging, osteoporosis, atheroma, asthma, joint diseases, and cancer^13^,^14^. ROS can cause oxidative stress in the inflammatory and apoptotic process, and are thus deleterious at high concentrations^15^.

Oxidative damage can suppress osteogenesis^16^. Osteoclasts are very sensitive to oxidative stress^17^,^18^,^19^. Low levels of ROS may stimulate osteoclast bone resorption during bone resorption and osteoclast differentiation^20^,^21^,^22^. However, beyond a certain threshold, chronic exposure of osteoclasts to elevated oxidative stress results in cytotoxic effects due to the increased oxidative damage of DNA, proteins, and lipids, which can then lead to apoptosis via the caspase-dependent pathway^23^. A recent study has also found that high levels of ROS inhibit human and mouse osteoclast differentiation^24^. However, the ROS-mediated apoptotic pathway is not fully understood. Thus, we hypothesized that ROS could promote apoptosis of osteoclast precursors and osteoclasts via intracellular signal pathways. The purpose of this study was, therefore, to investigate the ROS-mediated intracellular signal pathways in ZA-treated osteoclast precursors.

## Results

### ZA treatment induces apoptosis in monocytes, macrophages, and differentiated osteoclast-like cells

**Figure 1:**
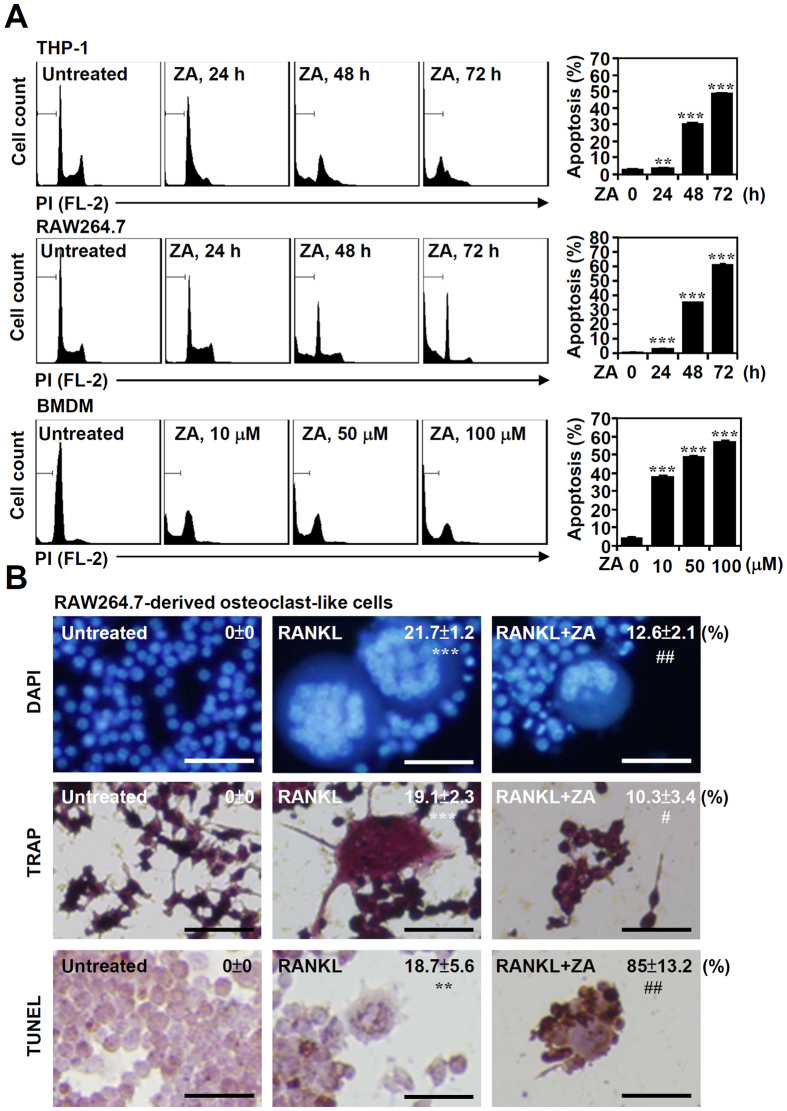
ZA treatment induces apoptosis in monocytes, macrophages, and differentiated osteoclast-like cells. (**A**) THP-1 and RAW 264.7 cells were treated with ZA (100 μM) for the indicated time. BMDMs were treated with different doses of ZA as indicated for 48 h. A representative histogram obtained from PI staining followed by flow cytometric analysis indicated the proportion of cells in the sub-G_1_ phase. The data are shown as the mean ± SD of three individual experiments. ***p < 0.001 compared to untreated cells. (**B**) RAW264.7 cells were pre-treated with RANKL for 6 days followed by ZA (100 μM) treatment for another 2 days. DAPI, TRAP, and TUNEL staining followed by fluorescent and light microscopic observation were used to detect the formation of osteoclast-like cells and cell apoptosis. A representative image obtained from three individual experiments is shown. TRAP staining followed by microscopic observation reveals the differentiation of osteoclasts, which are characterized by TRAP-positive multinucleated cells (nuclei > 3). The percentages of osteoclast-like cells and apoptotic cells are shown as the means ± SD. Scale bar = 50 μm. **p < 0.01 and ***p < 0.001 compared to untreated cells; ^#^*p* < 0.05 and ^##^*p* < 0.01 compared to RANKL treatment.

To investigate the effects of ZA, we used PI staining followed by flow cytometric analysis to determine the level of apoptosis in the osteoclast precursor cell lines. The results showed that ZA treatment induced apoptosis in mouse macrophage cell line RAW264.7 (murine leukemia virus transformed) and human monocytic cell line THP-1 (isolated from patient with acute monocytic leukemia), in a time-dependent manner (Fig. 1A, top and middle). Additionally, by using primary isolated bone marrow-derived macrophages (BMDMs), ZA induced dose-dependent cell apoptosis (Fig. 1A, bottom). To confirm the level of apoptosis in the differentiated osteoclasts after ZA treatment, RAW264.7 cells were pre-treated with RANKL for 6 days followed by ZA (100 μM) treatment for another 2 days. Fluorescent imaging of DAPI-based nuclear staining (Fig. 1B, top) and Tartrate-resistant acid phosphatase (TRAP) staining, an osteoclast marker, (Fig. 1B, middle) showed a decrease in the formation of RAW264.7-derived osteoclast-like cells following ZA stimulation. Further terminal deoxynucleotidyl transferase dUTP nick end labeling (TUNEL) staining revealed ZA-induced DNA fragmentation, a marker of cell apoptosis, in the RAW264.7-derived osteoclast-like cells (Fig. 1B, bottom). These results suggest that ZA induces apoptosis in monocytes, macrophages, and differentiated osteoclast-like cells.

### ZA treatment causes caspase activation during apoptosis of osteoclast precursors

**Figure 2:**
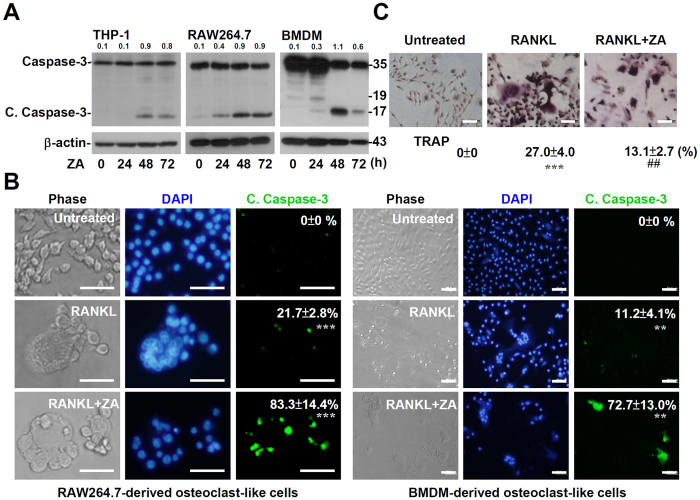
ZA treatment causes caspase-3 activation. (**A**) THP-1, RAW 264.7 cells, and BMDMs were treated with ZA (100 μM) for the indicated time. Western blotting analysis detected the expression of the caspase-3. β-actin was used as an internal control. One representative data set with an optical density from three individual experiments is shown. The relative ratios of cleaved caspase-3 (C. Caspase-3) to β-actin are shown. Full-length blots/gels are presented in Supplementary Figure 1. (**B**) RAW264.7 cells and BMDMs were pre-treated with RANKL for 6 days followed by ZA (100 μM) treatment for another 2 days. Immunostaining followed by fluorescence microscopy revealed the signals from cleaved caspase-3 staining (*green*). (**C**) TRAP staining of ZA-treated BMDM-derived osteoclast-like cells. A representative image obtained from three individual experiments is shown. For image analysis, DAPI (*blue*) was used for nuclear staining. Scale bar = 50 μm. We calculated the cell number of c. caspase-3-positive cells as percentages as compared with total cells per culture. The result is shown as the mean ± SD of triplicate cultures obtained from three individual experiments. ***p < 0.001 compared to untreated cells.

Cell apoptosis and survival are regulated by a variety of proteins^25^. Previous literature showed that caspase-3 participates in the differentiation, activity, and apoptosis of osteoclasts^26^,^27^. Therefore, we examined the role of caspases-3 in ZA-induced cell death in THP-1 cells, RAW264.7, and BMDMs. Western blotting confirmed the activation of caspase-3 in a time-dependent manner (Fig. 2A). While RANKL stimulation caused RAW264.7 cells and BMDMs to differentiate into mature osteoclast-like cells, immunostaining was used to determine the expression of cleaved caspase-3 in mature osteoclast-like cells. Activated caspase-3 was significantly (*p* < 0.001) increased after ZA treatment (Fig. 2B). TRAP staining confirmed the differentiation of osteoclast-like cells in RANKL-treated BMDMs while ZA treatment caused a significant decrease (*p* < 0.01) in the number of osteoclast-like cells (Fig. 2C). These results demonstrate that ZA treatment causes caspase-3 activation in monocytes, macrophages, and differentiated osteoclast-like cells.

### ROS mediate the apoptotic pathway in ZA-stimulated osteoclast precursors

**Figure 3:**
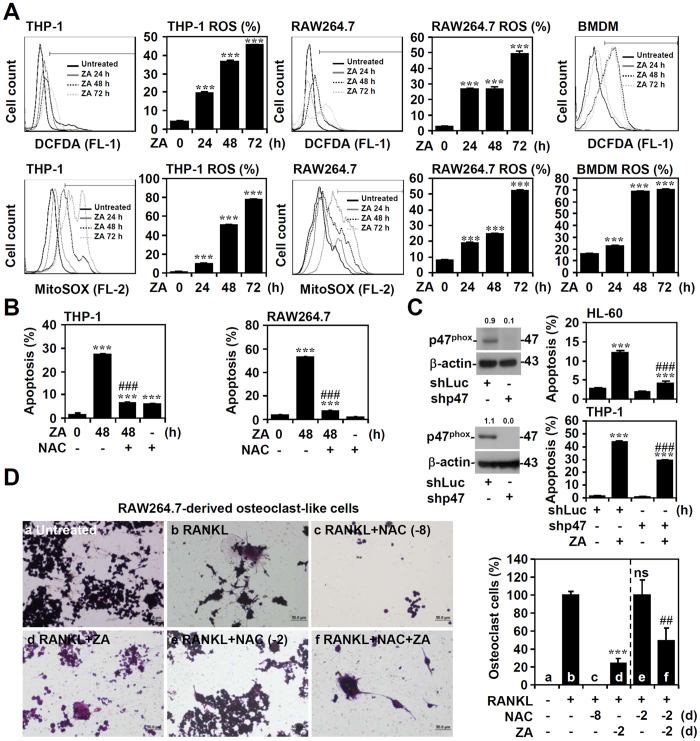
ZA treatment triggers ROS generation followed by ROS-mediated cell apoptosis. (**A**) The generation of ROS and mitochondrial ROS in ZA (100 μM)-treated THP-1, RAW264.7 cells, and BMDMs were determined using CM-H_2_DCFDA staining and MitoSOX staining followed by flow cytometry for the indicated time. The data are shown as the means ± SD from triplicate cultures. ***p < 0.001 compared to untreated cells. (**B**) THP-1 and RAW264.7 cells were treated with ZA (100 μM) in the presence of a ROS inhibitor NAC (20 mM) for 48 h. A PI-based flow cytometric analysis was used to measure apoptosis. ***p < 0.001 compared to untreated cells; ^###^*p* < 0.001 compared to ZA treatment. (**C**) Lentivirus-based shRNA was used to silence p47^phox^ in HL-60 and THP-1 cells, and protein expression was confirmed by Western blotting analysis for the indicated time. shLuc was used as the negative control. Full-length blots/gels are presented in Supplementary Figure 2. A PI-based flow cytometric analysis was used to measure apoptosis. One representative data set obtained from three individual experiments is shown in each experiment. The data are shown as the mean ± SD from three individual experiments. ***p < 0.001 compared to untreated cells; ^###^*p* < 0.001 compared to shLuc. (**D**) RAW264.7 cells were pre-treated with RANKL for 6 days followed by ZA (100 μM) in the presence of the ROS inhibitor NAC (20 mM) treatment for another 2 days. The number of differentiated osteoclast-like cells in the RANKL group was normalized to 100%. Scale bar = 50 μm. The data are shown as the mean ± SD of triplicate cultures obtained from three individual experiments. ***p < 0.001 compared to RANKL treatment; ^##^*p* < 0.01 compared to RANKL treatment with NAC. ns, not significant.

Previous literature showed that ZA may induce apoptosis via ROS production^28^,^29^. We next examined the involvement of ROS in the induction of apoptosis in ZA-treated THP-1, RAW264.7 cells, and BMDMs. CM-H_2_DCFDA, a chloromethyl derivative of H_2_DCFDA used as an indicator for ROS in cells, and MitoSOX, a novel fluorogenic dye specifically targeted to mitochondria in live cells, staining followed by flow cytometric analysis demonstrated that ZA induced a significant increase in the level of ROS in a time-dependent manner (*p* < 0.001) (Fig. 3A). Using PI staining followed by flow cytometric analysis, we found that pre-treating THP-1 and RAW264.7 cells with the ROS inhibitor *N*-Acetyl-L-cysteine (NAC) prior to ZA stimulation significantly reduced cell apoptosis compared to the ZA only treated groups (*p* < 0.001) (Fig. 3B). It is known that p47^phox^ plays an important role in NADPH oxidase and ROS generation^30^. Therefore, a lentiviral-based shRNA approach was used to silence p47^phox^. It is notable that shp47^phox^-transfected HL-60 and THP-1 cells showed a significant decrease in ZA-induced cell apoptosis (*p* < 0.001) (Fig. 3C). To examine the role of ROS in osteoclast maturation, TRAP staining followed by microscopic observation revealed that the osteoclasts differentiated, which was characterized by TRAP-positive multinucleated cells (nuclei > 3). RAW264.7 cells were pre-treated with RANKL followed by treatment with ZA and the ROS inhibitor NAC. By using TRAP staining, the number of differentiated osteoclast-like cells in the RANKL group was normalized to 100%. In the presence of NAC, we found a significant decrease in cell counts after ZA treatment (*p* < 0.01). In addition, we also found that osteoclasts did not form when the cells were co-treated with RANKL and NAC for 8 days (Fig. 3D). These results provide evidence that ROS generation facilitates osteoclast formation in osteoclast precursors and ZA-induced cell death in mature osteoclast-like cells.

### MAPKs are unable to promote apoptosis of osteoclast precursors

**Figure 4:**
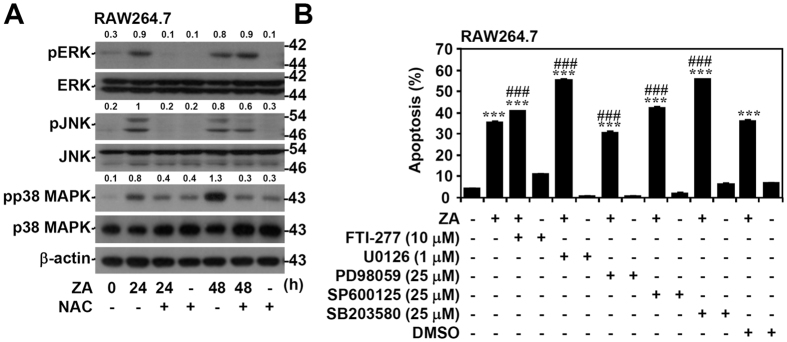
MAPK activation is not involved in ZA-induced cell apoptosis. (**A**) RAW264.7 cells were treated with ZA (100 μM) in the presence of the ROS inhibitor NAC (20 mM) for the indicated time. Western blotting detected the expression of phosphorylated ERK (pERK), ERK, phosphorylated JNK (pJNK), JNK, phosphorylated p38 MAPK (pp38 MAPK), and p38 MAPK. β-actin was used as an internal control. One representative data set with an optical density from three individual experiments is shown compared to the normalized control. Full-length blots/gels are presented in Supplementary Figure 3. (**B**) RAW264.7cells were treated with ZA (100 μM) in the presence of the farnesyl transferase (FTase) inhibitor FTI-277, the MEK inhibitors U0126 and PD98059, the JNK inhibitor SP600125, and the p38 MAPK inhibitor SB203580 for 48 h. DMSO was used as a negative control. A PI-based flow cytometric analysis was used to measure apoptosis. One representative data set obtained from three individual experiments is shown. The data are shown as the mean ± SD from three individual experiments. ***p < 0.001 compared to untreated cells; ^###^*p* < 0.001 compared to ZA treated cells.

Recently, studies showed that signaling molecules, such as MAPKs, JNK, and p38 MAPK, are essential for osteoclast differentiation. These molecules are also sensitive to ROS activation^31^,^32^. Thus, we examined the effects of MAPKs ERK, JNK, and p38 MAPK on ZA-induced apoptosis in RAW264.7 cells. Protein analysis indicated that ZA induced ERK, JNK, and p38MAPK phosphorylation 24 h post-stimulation. It is notable that the pharmacological treatment of cells with the ROS inhibitor NAC caused ERK, JNK, and p38 MAPK inactivation (Fig. 4A). These results suggest that ROS regulate ZA-induced activation of MAPKs. However, apoptotic analysis showed that treatment with the RAS inhibitor FTI 277, the MEK inhibitors PD98059 and U0126, the JNK inhibitor SP600125, or the p38 MAPK inhibitor SB203580 did not block ZA-induced RAW264.7 cell apoptosis (Fig. 4B). These results demonstrate that MAPKs are not required for ZA-induced cell apoptosis.

### Overexpression of Mcl-1 promotes cell survival

**Figure 5:**
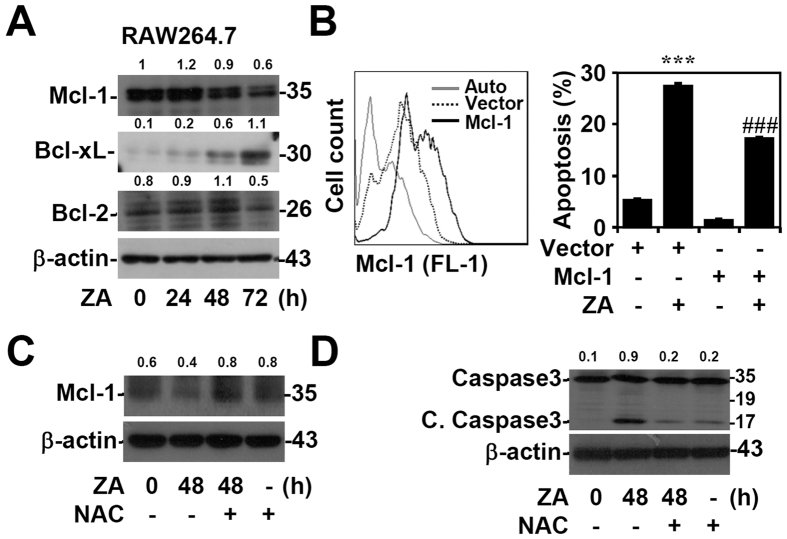
ZA treatment downregulates Mcl-1 expression by ROS to promote cell apoptosis. (**A**) RAW264.7 cells were treated with ZA (100 μM) for the indicated time. Western blotting determined the expression of Mcl-1, Bcl-xL, and Bcl-2. (**B**) Mcl-1 was overexpressed in RAW264.7 cell following transfection with pcDNA_3_-HA-Mcl-1, which was confirmed by a flow cytometry. The cells were then treated with ZA (100 μM) for 2 days. pcDNA_3_-HA was used as the vector control. A PI-based flow cytometric analysis was used to measure apoptosis. ***p < 0.001 compared to vector; ^###^*p* < 0.001 compared to vector with ZA treatment. RAW264.7 cells were treated with ZA (100 μM) in the presence of the ROS inhibitor NAC (20 mM). Western blotting analysis revealed the expression of Mcl-1 (**C**), cleaved caspase-3 (*C. Caspase*-*3*), and caspase-3 (**D**). β-actin was used as an internal control. One representative data set with the relative optical density from three individual experiments is shown. Full-length blots/gels are presented in Supplementary Figure 4.

We next investigated the effects of ZA on the expression of the Bcl-2 family of proteins, including the anti-apoptotic proteins Mcl-1, Bcl-2, and Bcl-xL in RAW264.7 cells. After ZA treatment, protein analysis showed that ZA caused an increase in Bcl-xL expression and a decrease in Mcl-1 expression (Fig. 5A). Regarding our previous study in which we explored the pro-survival role of increased Bcl-xL expression in ZA-treated cells^33^, this study further investigated the cause of cell apoptosis following a decrease in the levels of the Bcl-2 family of proteins. To further verify the role of Mcl-1 in ZA-induced apoptosis, a plasmid containing pcDNA-HA-Mcl-1 was transfected into RAW264.7 cells to overexpress Mcl-1, which significantly (*p* < 0.001) rescued ZA-induced apoptosis (Fig. 5B). We further examined the involvement of ROS in the apoptosis of ZA-treated RAW264.7 cells. Pharmacological inhibition of ROS by NAC reversed the ZA-induced downregulation of Mcl-1 (Fig. 5C) and also suppressed the activation of caspase-3 (Fig. 5D). These findings indicate that ROS may decrease Mcl-1 stability, which is involved in ZA-induced cell apoptosis.

### GSK-3β activation is required for ZA-induced Mcl-1 downregulation and cell apoptosis

**Figure 6:**
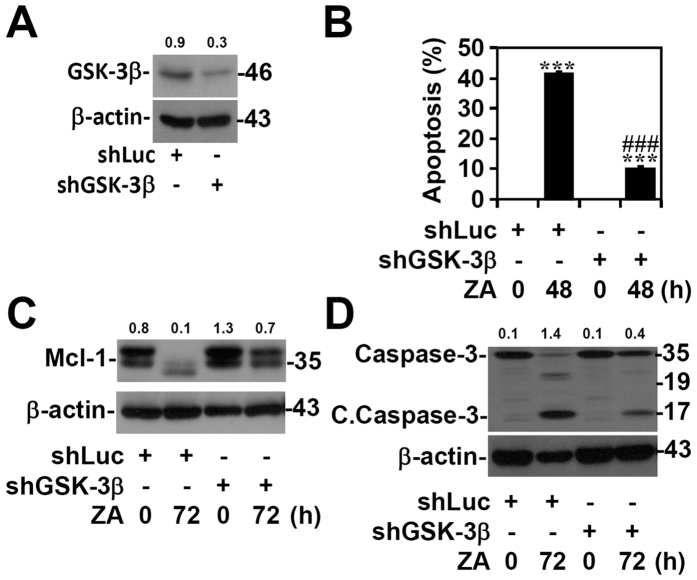
GSK-3β is required for ZA-induced Mcl-1 downregulation and cell apoptosis. (**A**) The expression of GSK-3β was silenced in RAW264.7 cells using a lentiviral-based shRNA. shLuc was used as the negative control. Western blotting analysis determined the expression of GSK-3β. (**B**) shLuc- or shGSK-3β-transfected RAW264.7 cells were treated with ZA (100 μM) for 2 days. The percentage of apoptotic cells was detected using PI-based flow cytometry. Western blotting analysis determined the expression of Mcl-1 (**C**), cleaved caspase-3 (*C. Caspase*-*3*), and caspase-3 (**D**) in shLuc- or shGSK-3β-transfected RAW264.7 cells after ZA treatment for 3 days. β-actin was used as an internal control. One representative data set with an optical density from three individual experiments is shown. Full-length blots/gels are presented in Supplementary Figure 5.

GSK-3β has been reported as a pro-apoptotic protein in various cell types^34^,^35^,^36^. A genetic approach was used to investigate the essential role of GSK-3β in ZA-induced apoptosis. Lentiviral-based shRNA knock-down of GSK-3β (Fig. 6A) significantly reduced ZA-induced apoptosis in RAW264.7 cells (*p* < 0.001) (Fig. 6B). Protein analysis showed that knock-down of GSK-3β reversed ZA-induced Mcl-1 downregulation (Fig. 6C) and caused a decrease in ZA-induced caspase-3 activation (Fig. 6D). These results indicate that ZA causes a GSK-3β-mediated decrease in Mcl-1 followed by activation of caspase-3 to facilitate cell apoptosis.

### Treatment of ZA induces a loss in mitochondrial transmembrane potential

**Figure 7:**
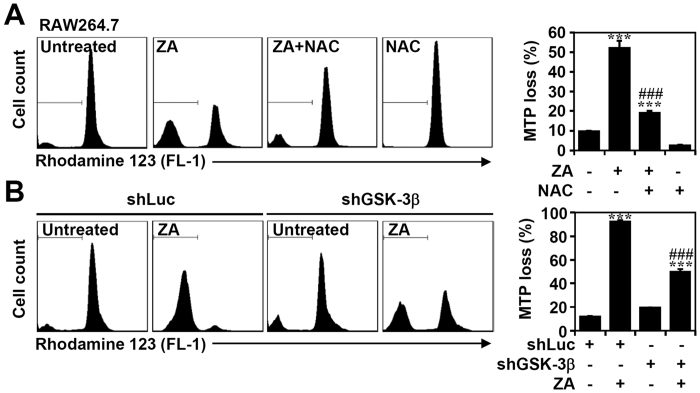
Inhibiting ROS and knock-down of GSK-3β decreases the ZA-induced loss of the MTP in RAW264.7 cells. (**A**) RAW264.7 cells were treated with ZA (100 μM) for 2 days in the presence of NAC (20 mM). (**B**) shLuc- or shGSK-3β-transfected RAW264.7 cells were treated with ZA (100 μM) for 2 days. MTP was determined using rhodamine 123 followed by flow cytometric analysis. The data are shown as the mean ± SD from three individual experiments. ***p < 0.001 compared to untreated cells; ^###^*p* < 0.001 compared to ZA treatment.

Loss of Mcl-1 results in mitochondrial dysfunction, which is typically required for apoptosis to occur^37^,^38^. To investigate the involvement of dysfunctional mitochondria in apoptosis, the ROS inhibitor NAC was used. The ZA-induced loss of MTP in RAW264.7 cells was detected by lipophilic, cationic, fluorochrome, rhodamine 123 staining followed by flow cytometric analysis, suggesting that ZA induces a mitochondria-dependent apoptotic pathway. Notably, inhibiting ROS using NAC blocks ZA-induced mitochondrial damage (Fig. 7A). Furthermore, lentivirus-based shRNA knock-down of GSK-3β also significantly (*p* < 0.001) reduced ZA-induced MTP loss in RAW264.7 cells (Fig. 7B). These results demonstrate that ZA induces ROS and GSK-3β activation followed by mitochondrial dysfunction.

## Discussion

**Figure 8:**
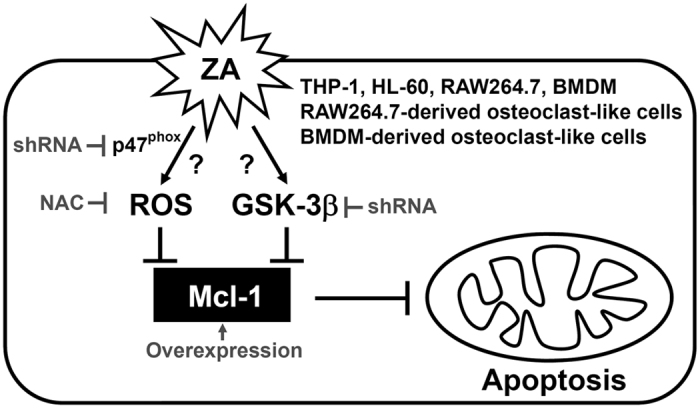
A hypothetic model of ZA-induced cell apoptosis.

The most important finding of this study is the identification of a hypothetic molecular signaling mechanism for ZA-induced cell apoptosis in osteoclast precursors. We found that ZA induces both ROS and GSK-3β activation followed by Mcl-1 downregulation to induce mitochondrial-related apoptosis (Fig. 8).

Osteoporosis is a silent disease but remains a major health problem. It is the most common metabolic bone disease. Osteoporosis-related fractures cause significant morbidity and mortality^39^,^40^. Administration of bisphosphonates is an effective therapy to reduce the vertebral and non-vertebral fracture risk in the treatment of osteoporosis^41^. ZA is a nitrogen-containing, third-generation, long-acting bisphosphonate for the treatment of osteoporosis and is given as an annual intravenous infusion^42^. However, some patients are less responsive to ZA treatment, and the mechanisms of resistance and apoptosis are still unclear. Our previous study found that osteoclast precursors develop a survival advantage against the pro-apoptotic effects of ZA via p38 MAPK-regulated GSK-3β inactivation, followed by β-catenin-mediated Bcl-xL expression^33^. In addition to the drug resistance pathway, a further understanding of the mechanism of ZA-induced apoptosis is required for the development of new strategies to improve the treatment of osteoporosis. In this study, we found that ROS could promote the apoptosis of osteoclast precursors by downregulating Mcl-1 expression.

The results of our investigation show that ZA treatment induces apoptosis in monocytes, macrophages, and differentiated osteoclast-like cells. Osteoclast apoptosis may be stimulated by extrinsic signals that activate death receptors on the osteoclast surface^43^ or caused by intrinsic signals that disrupt mitochondrial membrane integrity, which induces caspase-dependent apoptosis^44^. Basically, ZA treatment causes caspase-3 activation followed by cell death. The role of caspase-3 in osteoclast apoptosis is well-established in the previous literature^26^,^27^. Osteoclasts carry a number of mitochondria, which is the major source of ROS. An intrinsic apoptotic pathway plays an important role in ROS-induced osteoclast apoptosis^44^.

ROS act as a second messenger in cell signaling. ROS are both normal and also dangerous for cellular metabolism^45^,^46^. The generation of intracellular ROS is increased when RANKL binds to its receptor RANK on the cell surface of osteoclast precursors^22^. This process helps cells to maintain normal physiological functions. However, high levels of ROS can also cause oxidative stress and directly damage cells^47^. Cancer therapy, radiotherapy, chemotherapy, and photodynamic therapy can often promote ROS generation to kill tumor cells. In our study, we found ZA acts in a manner similar to chemotherapy agents. It triggers ROS generation followed by ROS-mediated cell apoptosis in osteoclast precursors and mature osteoclast-like cells.

The MAPK pathway is an important intracellular signaling chain. Previous studies in the literature have reported that this pathway may be activated following ROS activation^48^,^49^. One MAPK signaling molecule JNK is an essential protein in osteoclast development^31^. Activation of JNK is usually required for cell apoptosis^50^. ROS-mediated, prolonged JNK activation induces DNA damage and Mcl-1 downregulation. This pro-apoptotic signaling further triggers mitochondrial dysfunction, caspase activation, and cell apoptosis^51^. Furthermore, activation of p38 MAPK by ROS is essential in stress-induced injury^32^. p38 MAPK is reported to confer dual roles in both cell survival and cell death^52^. In our previously study^33^, we showed that p38 MAPK is expressed in osteoclast precursors and plays a pro-survival role following ZA treatment. In this study, we confirmed this result and further showed that MAPK activation is not involved in ZA-induced cell apoptosis (Fig. 7).

NADPH oxidase controls ROS generation through the assembly of PHOX subunits, including p91^phox^, p22^phox^, p47^phox^, and p67^phox^ 53. Our previous study showed that all-*trans* retinoic acid-induced apoptosis is mediated by an increase of NADPH oxidase/ROS to cause PI3K/AKT inactivation, GSK-3β activation, and Mcl-1 downregulation^36^. In this study, we silenced the NADPH oxidase subunit p47^phox^ using lentivirus-based shRNA and significantly reduced ZA-induced apoptosis. This result suggests that ZA induces NADPH oxidase-mediated ROS generation and further induces the apoptosis of osteoclast precursors. The mechanisms by which ZA-induced activation of NADPH oxidase need further investigation.

We found a decrease in Mcl-1 expression but an increase in Bcl-xL expression following ZA treatment. The increase in Bcl-xL expression may be involved in cell survival in response to ZA stimulation, which we previously reported^33^. The ZA-induced downregulation of Mcl-1 expression and further apoptosis is mediated by ROS, while overexpression of Mcl-1 reverses this phenomenon. Our previous study showed that Mcl-1 has an anti-apoptotic function by inhibiting Bax activation prior to mitochondrial damage^54^. This result also confirmed the anti-apoptotic effect of Mcl-1.

Our results also showed that knock-down of GSK-3β decreases ZA-induced Mcl-1 downregulation, mitochondrial damage, and apoptosis. The pro-apoptotic role of GSK-3β is due to its direct phosphorylation of Mcl-1, followed by the latter’s degradation via the ubiquitin/proteasome-dependent pathway^55^. However, the mechanism of crosstalk between ROS and GSK-3β activation is still unknown and requires further studies.

In conclusion, our study demonstrates that ZA treatment triggers ROS and GSK-3β activation, which further causes Mcl-1 downregulation and mitochondrial damage and results in apoptosis of osteoclast precursor cells. This finding helps to understand the use of ZA in the treatment of osteoporosis and develop new therapeutic strategies.

## Methods

### Cells, culture condition, reagents, and antibodies

The mouse macrophage cell line RAW264.7 (ATCC. TIB71) was routinely grown on plastic in Dulbecco’s modified Eagle’s medium (DMEM; Invitrogen Life Technologies, Rockville, MD) supplemented with 10% fetal bovine serum (FBS; Invitrogen Life Technologies), 50 U/ml penicillin, and 50 μg/ml streptomycin in a humidified atmosphere with 5% CO_2_ and 95% air at 37 °C. Human monocytic THP-1 cells (ATCC. TIB202) were routinely grown on plastic in RPMI 1640 medium (Invitrogen Life Technologies, Rockville, MD) supplemented with L-glutamine, 10% fetal bovine serum (FBS; Invitrogen Life Technologies), 50 U/ml penicillin, and 50 μg/ml streptomycin in a humidified atmosphere with 5% CO_2_ and 95% air at 37 °C. Human APL HL60 cells (ATCC. CCL240) were cultured in RPMI-1640 medium (Invitrogen Life Technologies, Carlsbad, CA, USA) supplemented with 10% heat-inactivated fetal bovine serum (FBS, Invitrogen Life Technologies) and maintained at 37 °C with 5% CO_2_. To obtain bone marrow-derived macrophages (BMDMs), bone marrow cells were isolated from 6-week old male C57BL/6J mice and resuspended in RPMI 1640 medium (Invitrogen Life Technologies, Gaithersburg, MD) with 10% FBS. Cells were induced with medium containing 10 ng/mL recombinant mouse M-CSF for further 6 days.

ZA, DAPI (4′,6-diamidino-2-phenylindole), rhodamine 123, NAC (N-ACETYL-L-CYSTEINE), propidium iodide (PI), Ras inhibitor FTI-277, JNK inhibitor SP600125 and MEK inhibitors U0126 and PD98059 were purchased from Sigma-Aldrich (St Louis, MO). The p38 MAPK inhibitor SB203580 and PKC inhibitor were obtained from Calbiochem (San Diego, CA). The recombinant mouse receptor activator of nuclear factor-kappa B ligand (RANKL) was purchased from PeproTech (Rocky Hill, NJ). Antibodies against caspase-3, Mcl-1, Bcl-xL, Bcl-2, phospho-ERK at Thr202/Tyr204, ERK, phospho-JNK at Thr183/Tyr185, JNK, phospho-p38 MAPK at Thr180/Tyr182, p38 MAPK, and GSK-3β were purchased from Cell Signaling Technology (Beverly, MA). The mouse monoclonal antibody specific for β-actin was purchased from Chemicon International (Temecula, CA), and the Alexa Fluor 488- and HRP-conjugated goat anti-rabbit were purchased from Invitrogen (Carlsbad, CA). All of the drug treatments were assessed for cytotoxic effects using cytotoxicity assays prior to use in the experiments.

### Osteoclast formation assay

RAW264.7 cells and BMDMs were plated in triplicate at a density of 4 × 10^3^ cells with α-MEM (Invitrogen Life Technologies, Gaithersburg, MD) supplemented with 10 ng/ml RANKL in 12-well tissue culture plates for 6 days. After RANKL treatment, the cells were treated with 100 μM ZA (Sigma-Aldrich). To determine osteoclast differentiation, the leukocyte acid phosphatase kit (Sigma-Aldrich) was used to stain TRAP according to the manufacturer’s instructions. The TRAP^+^ cells with multi-nuclei were defined as osteoclasts in this study. The percentage of osteoclast-like cell differentiation in the group treated with RANKL alone was defined as 100.

### Cell death assay

For cell cycle analysis, the cells were fixed with 70% ethanol in PBS at 4 °C for 30 min. After washing twice with PBS, the cells were stained with 40 μg/ml PI (Sigma-Aldrich) plus RNase for 30 min at room temperature. The cells were analyzed using flow cytometry (FACSCalibur; BD Bioscience) with excitation set at 488 nm and emission detected in the FL-2 channel (565–610 nm). The samples were analyzed using CellQuest Pro 4.0.2 software (BD Biosciences), and quantification was performed using FlowJo (Tree Star, Inc., Ashland, USA). Small cell debris was excluded by gating on a forward scatter plot. For PI staining, the levels of apoptosis were reported and gated as percentages of sub-G_1_. To observe nuclear condensation, DAPI (Sigma-Aldrich)-stained cells were observed using a fluorescence microscope (BX51; Olympus, Tokyo, Japan). To detect apoptosis in osteoclast-like cells, the cells were fixed with 3.7% formaldehyde for 30 min. Then, TUNEL staining was performed using an ApopTag Peroxidase *in situ* Apoptosis Detection Kit (Millipore, Billerica, MA) according to the manufacturer’s instructions. Several randomly selected areas per specimen were analyzed.

### Western blot analysis

The harvested cells were lysed with a buffer containing 1% Triton X-100, 50 mM of Tris (pH 7.5), 10 mM of EDTA, 0.02% NaN_3_, and a protease inhibitor cocktail (Roche Boehringer Mannheim Diagnostics, Mannheim, Germany). Following one freeze-thaw cycle, the cell lysates were centrifuged at 12,000 rpm at 4 °C for 20 min. The lysates were boiled in sample buffer for 5 min. The proteins were then subjected to SDS-PAGE and transferred to a PVDF membrane (Millipore, Billerica, MA) using a semi-dry electroblotting system. After blocking with 5% skim milk in PBS, the membranes were incubated with a 1/1000 dilution of primary antibodies at 4 °C overnight. The membranes were then washed with 0.05% PBS-Tween 20 and incubated with a 1/5000 dilution of horseradish peroxidase-conjugated secondary antibodies at room temperature for 1 h. After washing, the membranes were soaked in ECL solution (PerkinElmer Life Sciences, Inc., Boston, MA) for 1 min, and then exposed to film (BioMax; Eastman Kodak, Rochester, NY).

### Immunostaining

To detect the expression of the cleaved caspase-3 protein, the cells were fixed in 3.7% formaldehyde in PBS for 20 min. After two washes with PBS, the cells were stained with primary antibodies at 4 °C overnight. The next day, the samples were incubated with a mixture of Alexa 488-conjugated goat anti-rabbit IgG at room temperature for 1 h. DAPI was used for nuclear staining and was applied at room temperature for 20 min. The cells were washed with PBS and observed under a fluorescent microscope (BX51; Olympus, Tokyo, Japan).

### Determination of ROS production

ROS generation under ZA treatment was determined using 5-(and-6)-chloromethyl-2′, 7′-dichlorodihydrofluorescein diacetate, acetyl ester (CM-H_2_DCFDA; Invitrogen). The cells were incubated in 10 nM CM-H_2_DCFDA for 30 min at 37 °C and then analyzed using flow cytometry (FACSCalibur; BD Bioscience) with the excitation set at 488 nm. Emission was detected in the FL-1 (515–545 nm) channel, and the data were analyzed using FlowJo (Tree Star, Inc.). To detect mitochondrial ROS, the cells were stained with 5 μM MitoSOX Red (Invitrogen) for 10 min at 37 °C. After another washing with PBS, the cells were analyzed using flow cytometry (FACSCalibur; BD Bioscience) with the excitation set at 488 nm and the emission detected in the FL-2 channel (565–610 nm). The samples were analyzed using CellQuest Pro 4.0.2 software, and quantification was performed using FlowJo.

### Mitochondrial functional assay

To evaluate mitochondrial damage, the loss of mitochondria transmembrane potential (MTP) was assayed using rhodamine 123 (Sigma–Aldrich) and then analyzed using flow cytometry (FACSCalibur) with the excitation set at 488 nm. The emission was detected in the FL-1 channel (515–545 nm). The samples were analyzed using CellQuest Pro 4.0.2 software, and quantification was performed using FlowJo software. Small cell debris was excluded by gating on the forward scatter plot.

### Lentiviral-based RNA interference transfection

p47^phox^ knock-down in THP-1 and HL-60 cells and GSK-3β knock-down in RAW264.7 cells were performed using lentiviral transduction to stably express short hairpin RNAs (shRNA). A non-targeting shRNA control vector (shLuc; TRCN0000072247) and shRNA constructs targeting human p47^phox^ no. 1 (shNCF1; TRCN0000256331 containing 5′-CCATTGCCAACTACGAGAAGA-3′) and shRNA constructs targeting mouse GSK-3β (shGSK3β-; TRCN0000012615 containing 5′- CATGAAAGTTAGCAGAGATAA -3′) were obtained from the National RNAi Core Facility located at the Institute of Molecular Biology/Genomic Research Center, Academia Sinica, Taiwan. The lentivirus was obtained from the RNAi Core of Research Center of Clinical Medicine, National Cheng Kung University Hospital. 293T cells were co-transfected with 5 μg of packaging plasmid (pCMVΔR8.91), 0.5 μg of the envelope plasmid (pMD.G), and 5 μg of pLKO.1 shRNA using Lipofectamine 2000 (Invitrogen) for 6 h. After 24 h, the supernatants containing viral particles were harvested and filtered through 0.45 mm filters. The cells were infected according to a previously described protocol^56^. In brief, RAW264.7, THP-1, and HL-60 cells were transduced with lentivirus at an appropriate multiplicity of infection in complete growth medium supplemented with 8 μg/ml polybrene (Sigma-Aldrich). After transduction for 24 h and puromycin (Calbiochem, San Diego, CA) selection for 1 week, protein expression was monitored using western blot analysis.

### Mcl-1 overexpression

For Mcl-1 overexpression, RAW264.7 cells were transfected using Turbofect (Thermo Scientific) with pcDNA3-HA and pcDNA3-HA-Mcl-1 plasmids in serum-free medium and incubated for 20 min at room temperature. The transfected cells were cultured in DMEM growth medium for 24 h, and Mcl-1 expression was monitored using flow cytometry analysis.

### Statistical analysis

The values are expressed as the mean ± standard deviation (SD). The groups were compared using Student’s two-tailed unpaired t-test or one-way ANOVA analysis followed by the Dunnet’s post-hoc test when appropriate. Statistical significance was set at *p* < 0.05.

## Additional Information

**How to cite this article**: Tai, T.-W. *et al*. Reactive oxygen species are required for zoledronic acid-induced apoptosis in osteoclast precursors and mature osteoclast-like cells. *Sci. Rep.* **7**, 44245; doi: 10.1038/srep44245 (2017).

**Publisher's note:** Springer Nature remains neutral with regard to jurisdictional claims in published maps and institutional affiliations.

## Supplementary Material

Supplemental Information
