# Supplementary material for: Reactive oxygen species are required for zoledronic acid-induced apoptosis in osteoclast precursors and mature osteoclast-like cells
Source: Sci Rep. 2017 Mar 10;7:44245. doi: 10.1038/srep44245 (PMC5345019; doi:10.1038/srep44245)

Reactive oxygen species are required for zoledronic acid-induced apoptosis in osteoclast precursors and mature osteoclast-like cells

Ta-Wei Tai, Ching-Yu Chen, Fong-Chin Su, Yuan-Kun Tu, Tsung-Ting Tsai, Chiou-Feng Lin, and I-Ming Jou

Figure S1 Protein assay for Figure 2.
Figure S2 Protein assay for Figure 3.
Figure S3 Protein assay for Figure 4.
Figure S4 Protein assay for Figure 5.
Figure S5 Protein assay for Figure 6.

Tai *et al.* Supplemental Figure 1

For Figure 2A

THP-1

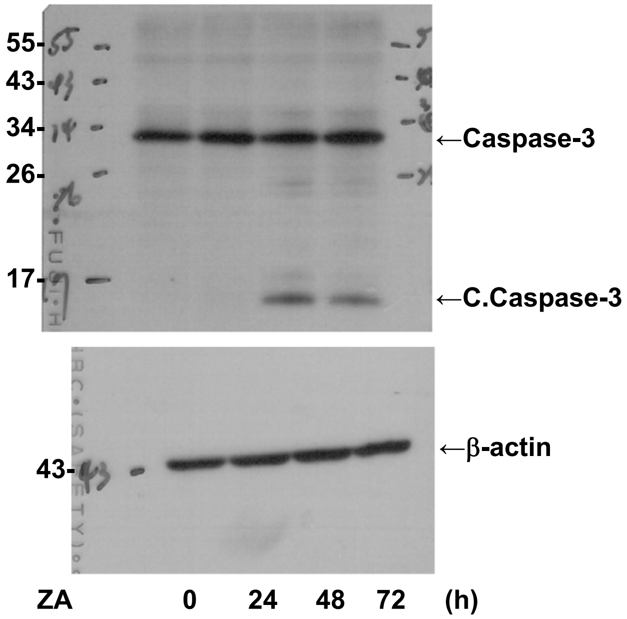

RAW264.7

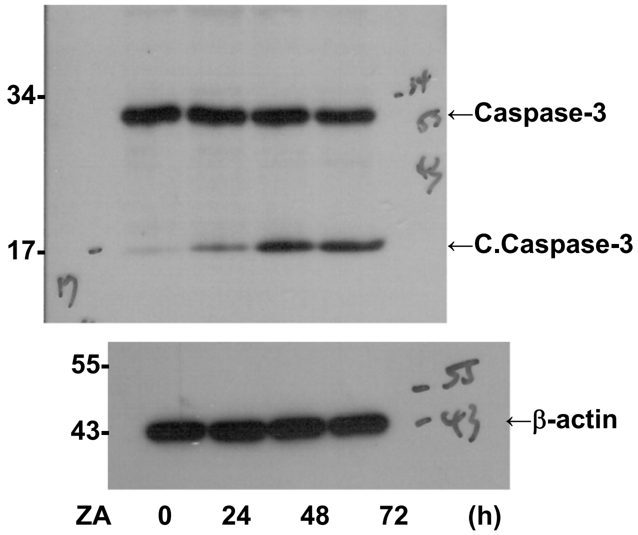

BMDM

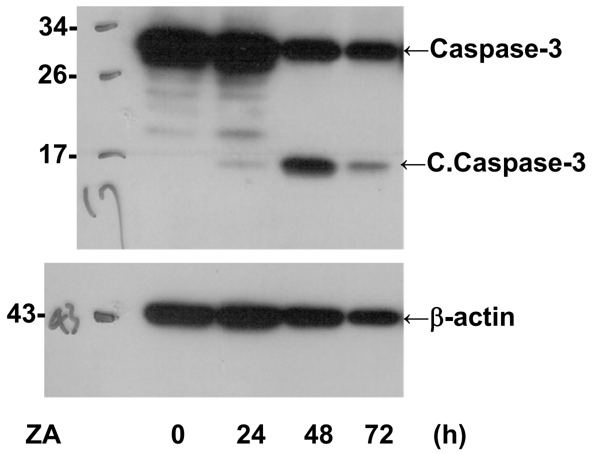

Tai *et al.* Supplemental Figure 2

For Figure 3C

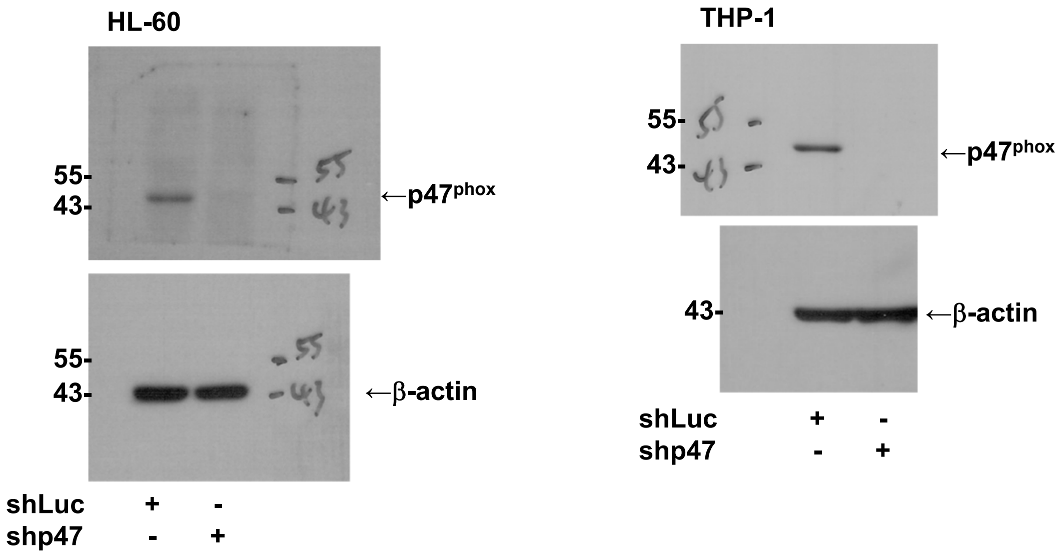

Tai *et al.* Supplemental Figure 3

For Figure 4A

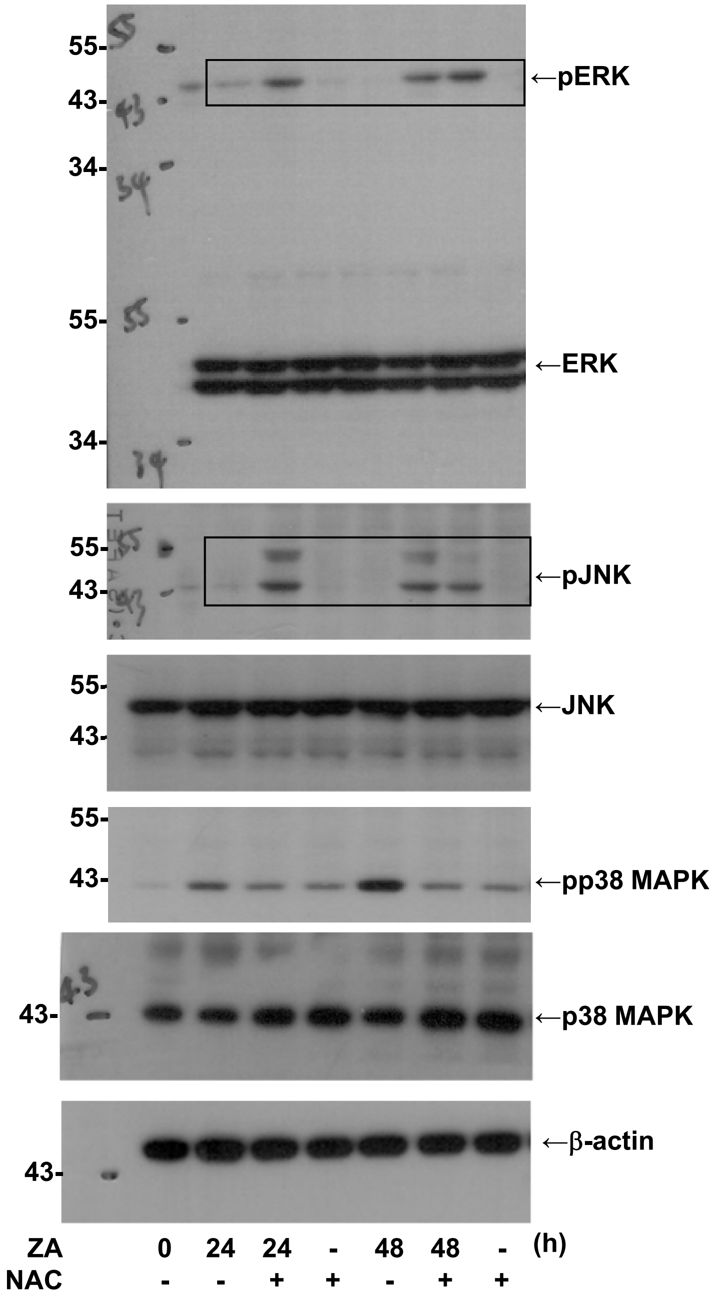

Tai *et al.* Supplemental Figure 4

For Figure 5A

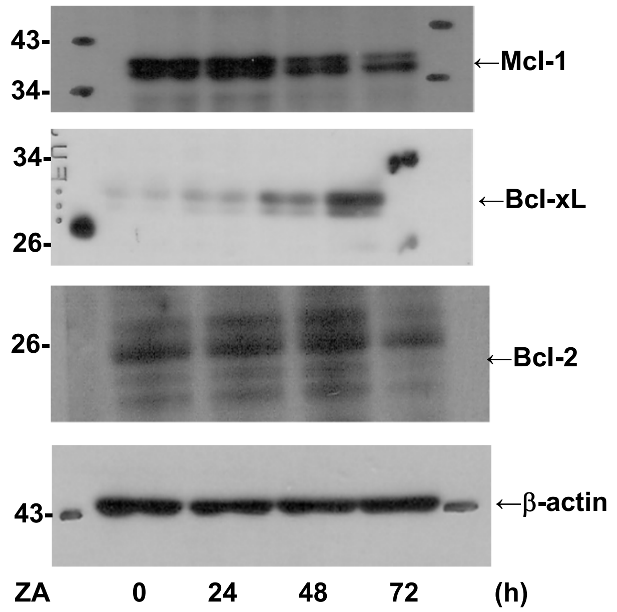

For Figure 5C

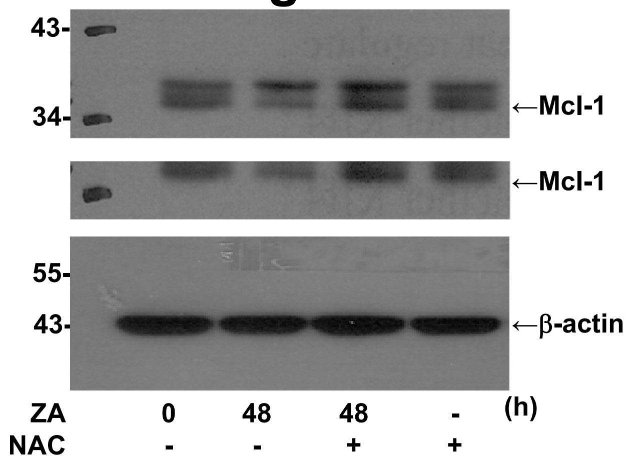

For Figure 5D

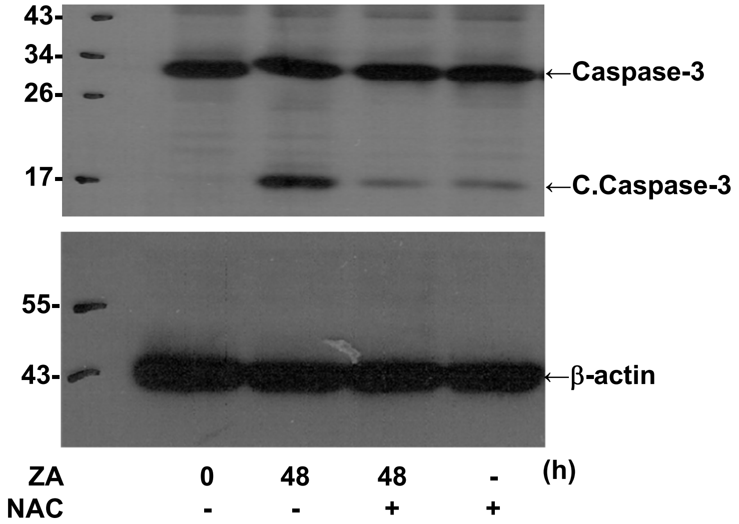

Tai *et al.* Supplemental Figure 5

For Figure 6A

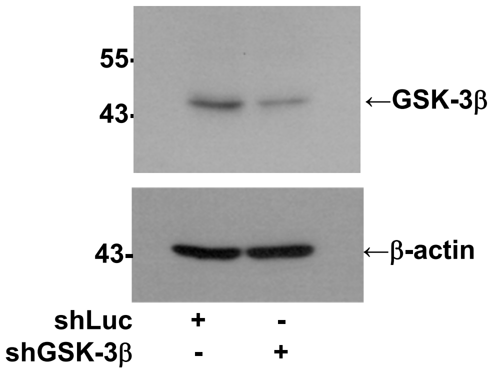

For Figure 6C

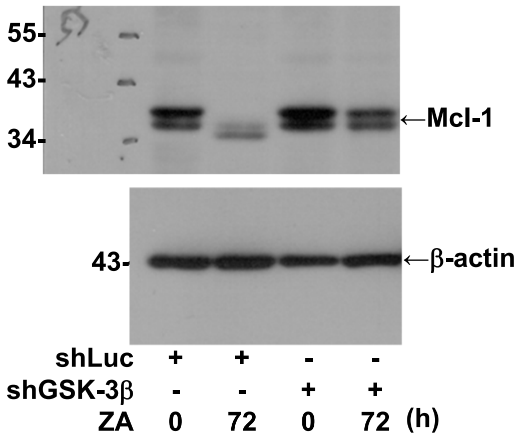

For Figure 6D

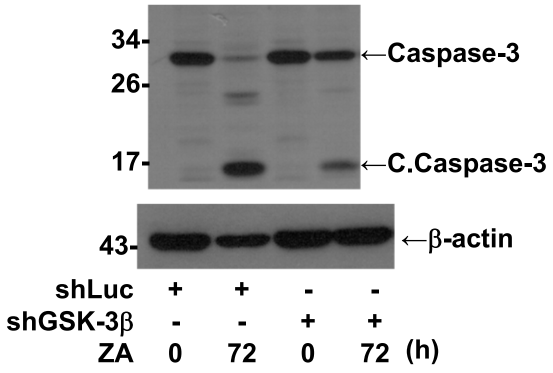

Supplement: Supplemental Information [file srep44245-s1.pdf]
